# Age-induced aortic modifications are accompanied by alterations in the antioxidant defense system in female rats

**DOI:** 10.3389/fmed.2023.1283302

**Published:** 2023-11-24

**Authors:** Sabahat Binte Asad, Xin Qian, Jiao Wang, Wajeeha Asad, Qiang Gao, Yang Cao, Yujia Huang, Yousef A. Bin Jardan, Tawaf Ali Shah, Gezahign Fentahun Wondmie, Mohammed Bourhia, Chunmei Lu, Hui Zhu

**Affiliations:** ^1^Department of Physiology, Harbin Medical University, Harbin, China; ^2^Department of Microbiology, University of Karachi, Karachi, Pakistan; ^3^Department of Pharmaceutics, College of Pharmacy, King Saud University, Riyadh, Saudi Arabia; ^4^Department of Agriculture Engineering and Food Science, Shandong University of Technology, Zibo, China; ^5^Department of Biology, College of Science, Bahir Dar University, Bahir Dar, Ethiopia; ^6^Department of Chemistry and Biochemistry, Faculty of Medicine and Pharmacy, Ibn Zohr University, Laayoune, Morocco; ^7^Laboratory of Medical Genetics, Harbin Medical University, and The Key Laboratory of Preservation of Human Genetic Resources and Disease Control in China, Chinese Ministry of Education, Harbin, China

**Keywords:** aging, blood pressure, aorta, antioxidant defense system, NRF2 pathway, NRF2/HO-1 pathway

## Abstract

**Introduction:**

Aging leads to significant structural and functional changes in blood vessels, which disrupt their normal function and impact cardiovascular health. Current research is actively exploring the NRF2 antioxidative pathway, recognizing its role in protecting cells by preserving their antioxidant defenses against damage. However, there has been limited exploration into the role of the NRF2 pathway in vascular aging. The primary objective of this study was to determine whether age-related changes in the aorta are associated with variations in the baseline levels of antioxidant enzymes, with a particular emphasis on how the NRF2 pathway operates in the aortic wall.

**Methods:**

A group of healthy aging female SD rats was compared with their younger counterparts. Various assessments were conducted, including measuring blood pressure, analyzing serum lipid profiles, examining aortic tissue, and assessing the expression of antioxidant enzymes.

**Results:**

The results revealed significant differences in both blood pressure and serum lipid levels between the aged and younger rats. The examination of the aorta in older rats showed structural alterations, increased apoptosis, and the accumulation of fatty deposits. In the older rats, levels of SOD-1 (superoxide dismutase) and GSS (glutathione synthetase) were lower, whereas NRF2, KEAP-1 (Kelch-like ECH-associated protein 1), and HO-1 (Heme oxygenase 1) were higher.

**Discussion:**

This study advances our understanding of how aging affects the antioxidant system in blood vessels, particularly in relation to the regulation of the NRF2/HO-1 pathway in the aorta. These findings suggest that targeting the NRF2/HO-1 pathway could present anovel therapeutic approach for addressing age-related vascular issues.

## 1 Introduction

Aging is an inevitable aspect of life, representing a natural process of becoming older. It entails the progressive structural and functional deterioration of almost all tissues, leading to a gradual decline in the body’s ability to maintain homeostasis and ultimately resulting in natural death (1). Vascular aging poses the highest risk for cardiovascular pathologies, and the aorta is considered a sensitive organ affected by aging (2). Even with non-pathological aging, the aorta undergoes many structural distortions, such as an enlarged lumen, intimal-medial thickening (vascular remodeling), increased collagen, decreased elastin content, and thin, fragmented elastic lamina in both humans and animals (3, 4). In addition, age-associated gender-based differences in aortic structure and function have also been discovered (5).

Previous studies have highlighted that the rate of apoptosis increases with aging in many tissues and organ systems, including the cardiovascular system, endocrine system, immune system, and nervous system (6–8). Earlier, aging studies have documented the link between aortic stiffness and elevated blood pressure (9). Similarly, age-related increases in cholesterol levels have also been reported. With aging, blood lipid contents, especially LDL levels, increase, contributing to the accumulation of lipids in the vascular tissues and resulting in an atherogenic burden (10).

Oxidative stress is a hallmark of aging, resulting from an imbalance in the oxidant/antioxidant equilibrium. Aerobic organisms possess a robust cellular defense system to protect cells from oxidative damage (11–14). Under typical physiological conditions, there exists an equilibrium between oxidant production and their elimination by the antioxidative system, preventing oxidative stress and maintaining homeostasis (15). The antioxidant defense system plays a central role in cellular protection by eliminating harmful oxidants and preventing cells from oxidative damage (15, 16). Antioxidants are scavenger molecules that can slow or prevent the oxidation process by neutralizing the deleterious effects of reactive oxygen species (ROS) (16). The antioxidant capacity comprises first-line defense antioxidants, such as superoxide dismutase (SOD-1), catalase (CAT), the glutathione system, phase II detoxifying enzymes like NAD(P)H: quinone oxidoreductase (NQO-1) and heme oxygenase-1 (HO-1), and various cytoprotective signaling pathways, such as the NRF2 signaling pathway (11).

Superoxide (SOD-1) rapidly converts superoxide anion (O_2_^−^) to hydrogen peroxide (H_2_O_2_) and oxygen (O_2_). Subsequently, hydrogen peroxide is decomposed into water and oxygen by the catalase (CAT) enzyme (15). Glutathione synthetase (GSS) is a pivotal contributor to the glutathione synthesis pathway. GSS indirectly plays an antioxidant role by mediating the 2nd step in the synthesis of GSH (12). Recently, the cellular defensive NRF2 signaling pathway has received wide attention for its protective role in various chronic and age-related pathologies (7). NRF2 is a redox-sensitive transcription factor that facilitates the transcription of many antioxidants by binding to a particular antioxidant response element (ARE) in the gene’s promoter. Under normal conditions, NRF2 activity is tightly regulated by its cytoplasmic inhibitor KEAP-1 (17, 18). During oxidative stress, KEAP-1 undergoes conformational changes. Consequently, NRF2 dissociates from KEAP-1, translocates into the nucleus, and regulates cytoprotective phase II detoxifying enzymes such as NQO-1 and HO-1 (18).

Numerous aging studies report that the decline in antioxidant capacity results in oxidative stress-related cellular damage in various tissues (11). However, there is currently insufficient documentation regarding the basal regulation of the NRF2 signaling pathway and its cytoprotective effects on vascular aging. This study was designed to investigate whether age-associated aortic modifications are associated with alterations in the basal expression of antioxidant enzymes, with a focus on the NRF2 pathway and its regulation in the aortic wall. In this context, we evaluated blood pressure, serum lipid levels, aortic histology, and the expression of various antioxidant proteins to better understand their correlation with the complexity of the aging process in the aortic wall.

## 2 Materials and methods

### 2.1 Animals

Twenty female Sprague–Dawley (SD) rats were obtained from the animal center at Harbin Medical University. The rats were divided into two groups, with 10 rats in each group: healthy young rats (3 months old, weighing 180–220 g) and aging rats (24 months old, weighing 352–554 g). Physiological aging symptoms were confirmed through vaginal smears, which showed irregularities in the estrous cycle and low levels of senile estrogen (19). All rats were kept under controlled conditions, including a temperature range of 22°C–25°C, humidity maintained at 50% ± 10%, and a 12-h dark/light cycle. They were provided with a standard chow diet *ad libitum* and had free access to water. All experimental protocols used in this research received approval from the Institutional Animal Care and Use Committee (IACUC) at Harbin Medical University and the Institute of Laboratory Animal Science of China (A5655-01). The animals were handled in accordance with the U.K. Animals (Scientific Procedures) Act, 1986, and associated guidelines, as well as the EU Directive 2010/63/EU for animal experiments.

### 2.2 Blood pressure

A week before sacrificing the animals, blood pressure was non-invasively measured in conscious rats using a tail and cuff detector (BP-2010E, Softron Biotechnology Ltd., China). In brief, the rats’ tails were heated to 37°C for 10 min, and a cuff with a pneumatic pulse sensor was placed around each rat’s tail. Systolic, diastolic, and pulse pressure measurements were recorded for each rat and then averaged.

### 2.3 Sample collection

All animals were anesthetized with an intraperitoneal injection of 10% chloral hydrate and then euthanized. Blood samples were collected, placed at 4°C for 1 h, and subsequently centrifuged at 3,000 rpm at 4°C for 15 min. The collected serum samples were stored at −80°C for further evaluation. The thoracic aorta was excised, and all adventitial fats were removed. A part of the aorta was fixed in 4% paraformaldehyde and embedded in paraffin, while the remaining tissues were flash-frozen in liquid nitrogen and stored at −80°C for further analysis.

### 2.4 Serum lipid analysis

Serum levels of triglyceride (TG), total cholesterol (TC), low-density lipoprotein (LDL), and high-density lipoprotein (HDL) were assessed using the Triglyceride, Total cholesterol, Low-density lipoprotein cholesterol, and High-density lipoprotein cholesterol assay kits following the manufacturer’s protocol (Nanjing Jiancheng Bioengineering Institute, China).

### 2.5 Histological analysis by H&E staining

The paraffin-embedded tissues were cross-sectionally cut into 5 μm thick sections using a Leica RM 2016 rotator microtome. Following a previously described protocol, the paraffin sections were then deparaffinized and rehydrated before being stained with hematoxylin and eosin (H&E) (20). The H&E-stained slides were observed under a light microscope, and photographs were captured using the M8 Microscope & Scanner (Precipoint, Germany). The images were processed using ViewPoint BETA v1.0.0.0 software. Aortic intimal-medial thickness (IMT) was calculated using ImageJ (21). In brief, the image was opened in ImageJ, and the scale was set. Next, a line was drawn across the object using the ‘Straight Line’ tool. The length of the line was then measured by selecting ‘Analyze’ and ‘Measure.’ Multiple measurements were taken from different points in four H&E-stained images from each group. The average thickness was calculated by summing the measurements and dividing by the count of measurements.

### 2.6 Nuclear morphological analysis by Hoechst 33258 staining

Aortic tissues were stained with Hoechst 33258 fluorescent dye (Beyotime, China) to observe apoptotic nuclear morphological changes. Briefly, the paraffin sections of aortic tissues were deparaffinized in xylene, rehydrated in gradient alcohol, washed twice with cold PBS, and then incubated with Hoechst 33258 for 10 min at room temperature in the dark. Subsequently, the sections were washed again in PBS and examined under a Nikon inverted fluorescence microscope (Japan, Ti series). Photographs were taken for further quantitative analysis. The analysis of cell nuclear morphology and nuclei quantification was conducted using ImageJ software (22). In brief, the images were opened in ImageJ, and segmentation was achieved through the application of the “Threshold” tool (“Image” → “Adjust” → “Threshold”). To address overlapping nuclei, the “Watershed” function (“Process” → “Binary” → “Watershed”) was employed. Results, encompassing nuclei count and critical measurements, were accessed via “Analyze” → “Analyze Particles.” The percentage of apoptotic nuclei was calculated by comparing the number of nuclei displaying apoptotic morphology to the total number of nuclei in four images from each group.

### 2.7 Oil Red O staining

To examine the aortic lipid contents, freshly frozen aortic tissue samples from both groups were stained with Oil Red O (ORO; Nanjing Jiancheng Bioengineering Institute, China), as previously described (23). The ORO-stained slides were examined under a light microscope, and photographs were taken using the M8 Microscope and scanner (Precipoint, Germany). The percentage of ORO staining intensity in four samples from each group was quantified and compared in both groups using ImageJ software (21). In brief, the images were opened in ImageJ, and calibration was achieved by inputting the necessary calibration data in the “Properties” section of the software. The thresholding process, executed under “Adjust” > “Threshold,” was applied consistently to ensure uniform settings across all images. After thresholding, a binary image was generated through “Binary” > “Make Binary,” and the “Analyze Particles” function was employed for parameter measurements. Key metrics, including particle count, area, and circularity, were determined to quantify lipid droplets.

### 2.8 Western blotting

The frozen aortic tissues were lysed with RIPA buffer mixed with PMSF, and total protein extracts were obtained by centrifuging at 14,000 rpm for 15 min. Total protein concentration was calculated using a 96-well plate Bicinchoninic Acid (BCA) protein assay kit (Thermo Scientific, United States). A total of 20 μg of protein was electrophoretically fractionated by 10% SDS-PAGE and transferred to a polyvinylidene difluoride (PVDF) membrane using a Trans-Blot SD semi-dry transfer cell (Bio-Rad Laboratories, Richmond, Calif.). The membranes were blocked with 5% non-fat milk dissolved in Tris-buffered saline with 0.1% Tween 20 for 2 h. Next, membranes were incubated with the following primary antibodies: β-actin (1:2,000 dilution, Santa Cruz, United States), SOD-1 (1:1,000 dilution, Santa Cruz, United States), CAT (1:1,000 dilution, Santa Cruz, United States), GSS (1,1,000 dilution, Santa Cruz, United States), NRF2 (1:200 dilution, Abcam, United Kingdom), KEAP-1 (1,200 dilution, Abcam, United Kingdom), NQO1 (1:500 dilution, Abcam, United Kingdom), and HO-1 (1,500 dilution, Abcam, United Kingdom), which were incubated overnight at 4°C. The following day, membranes were incubated with horseradish peroxidase (HRP)-conjugated goat anti-rabbit IgG antibody (Santa Cruz Biotechnology, Santa Cruz, CA, United States) for 1 h at room temperature. The blotted proteins were visualized using Pierce ECL Western Blotting Substrate (Engreen Biosystem, China). The relative density of the bands was analyzed using ImageJ software (21).

### 2.9 Immunohistochemistry

The Paraffin-embedded sections of the aorta were deparaffinized and rehydrated, then treated with 0.3% H_2_O_2_ to block endogenous peroxidase activity. After blocking nonspecific reactions with goat serum, the primary rabbit polyclonal antibodies of SOD-1 (1,500 dilution, Santa Cruz, United States), CAT (1:500 dilution), GSS (1,500 dilution), NRF2 (1:200 dilution), KEAP-1 (1,200 dilution,), NQO1 (1:200 dilution), and HO-1 (1,200 dilution) were added and incubated for 12 h at 4°C. The following day, after rinsing with PBS, the sections were incubated with biotinylated goat antibody to rabbit-rat IgG at 37°C for 1 h and stained with DAB chromogenic and counterstained with hematoxylin. IHC-stained slides were examined under a light microscope, and photographs were taken using the M8 Microscope and scanner. The Immunohistochemistry (IHC) staining intensity of each IHC image was quantified using ImageJ software with the IHC profiler plugin (24). In brief, The IHC Profiler plugin was installed in ImageJ, and IHC stained images were opened within ImageJ. The IHC Profiler plugin was initiated by navigating to ‘Plugins’ > ‘IHC Profiler.’ In the plugin dialog, the image was selected, and custom ROIs were specified. This analysis involved pixel counting, assessing the percentage contributions, and assigning semiquantitative scores to our sections (categorized as positive or low positive). IHC optical density scores are determined and quantified on a scale from 1 to 4 based on an algebraic formula recommended by Seyed Jafari et al. (25). Subsequently, we calculated and compared the mean optical density values.

### 2.10 Statistical analysis

All results in this study were expressed as mean ± SD. For the statistical analysis, the student’s t-test was applied for the group comparisons and was evaluated by GraphPad Prism version 6.0c (GraphPad, La Jolla, CA) (26). *p* < 0.05 was considered statistically significant.

## 3 Results

### 3.1 Aging increases blood pressure in rats

**Figure 1:**
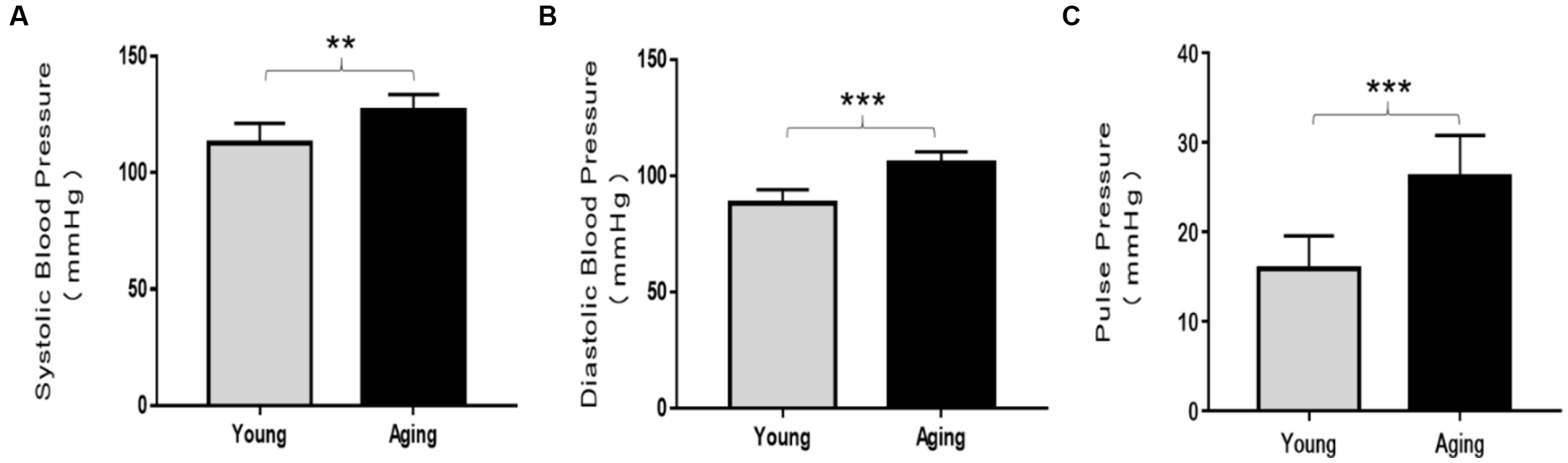
Comparison of blood pressure analysis between young and aging rats. In aging rats, systolic blood pressure (SBP), diastolic blood pressure (DBP), and pulse pressure (PP) significantly increased compared to the young rats. **(A)** Systolic blood pressure ^**^p < 0.01. **(B)** Diastolic blood pressure ^***^p < 0.001, and **(C)** pulse pressure ^***^p < 0.001 vs. young rats. Data represent the mean ± SD of 10 rats.

Systolic blood pressure (SBP), diastolic blood pressure (DBP), and pulse pressure (PP) were analyzed and compared between young and aging rats. SBP, DBP, and PP were significantly elevated in aging rats compared to those of young rats (Figures 1A–C). These results provide evidence that blood pressure increases with physiological aging.

### 3.2 Aging regulates serum lipid profile

**Figure 2:**
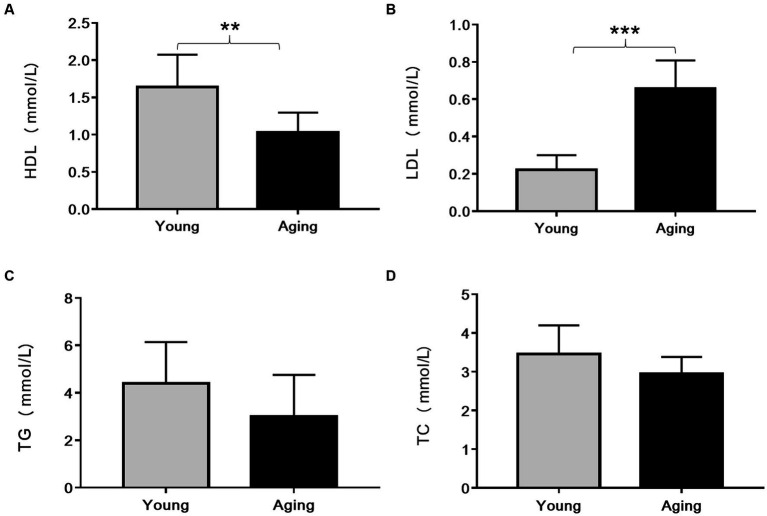
Comparison of serum lipid profile (mmol/L) between young and aging rats. Relative to young rats, **(A)** Serum HDL levels significantly decreased ^**^p < 0.01 whereas **(B)** Serum LDL levels significantly increased in aging rats ^***^p < 0.001. No significant differences were found in **(C)** TG and **(D)** TC levels between young and aging groups. Data represent the mean ± SD (*n* = 10 rats). HDL, High-density lipoprotein; LDL, Low-density lipoprotein; TG, Triglyceride; TC, Total cholesterol.

Compared to young rats, serum HDL levels significantly decreased, whereas LDL levels remarkably increased in the aging rats (Figures 2A,B). Although the total cholesterol and triglyceride levels slightly decreased in aging rats, they were not statistically significant (Figures 2C,D).

### 3.3 Aging alters the histology of the aorta

**Figure 3:**
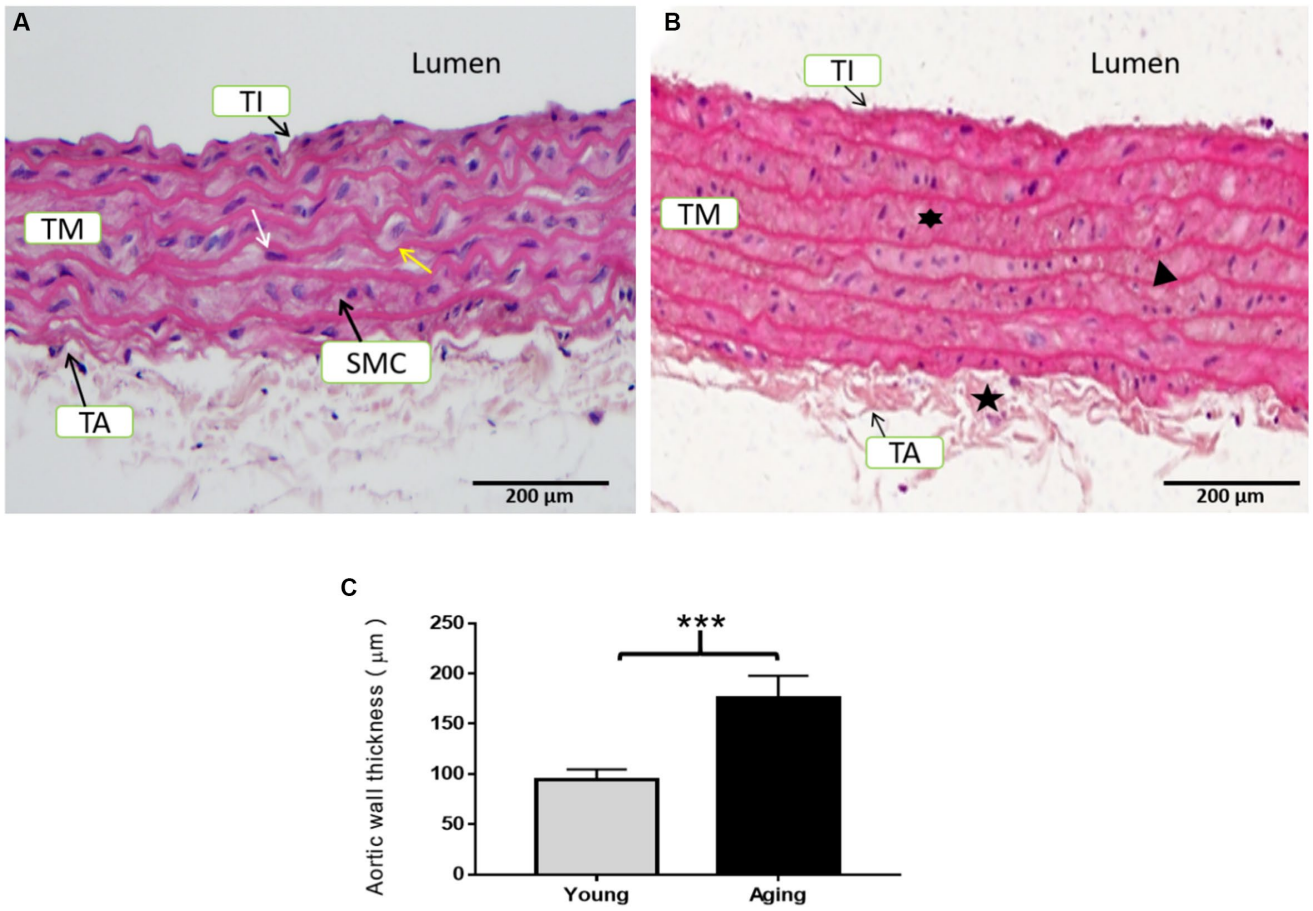
Representative H & E staining of the aorta’s cross-sectional tissue slices in young and aging rats. **(A)** Histological examination of the aorta from young rats displayed normal morphology. **(B)** Histological examination of the aorta from aging rats showed degenerative morphological modifications in all three layers. Scale bar = 200 μm. **(C)** Aortic wall thickness was significantly increased in aging rats ^***^p < 0.001 vs. young rats (*n* = 3). TI, Tunica intima; TM, Tunica media; TA, Tunica adventitia; SMC, Smooth muscle cells; IMT, Intimal-medial thickness; white arrow, internal elastic lamina; yellow arrow, nucleus of smooth muscle cells, 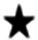 = fibrosed tunica adventitia, 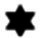 = proliferation of smooth muscle cells, 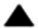 = thin and fragmented elastic lamina.

Histological examination of the aorta from young rats revealed typical morphology. The tunica intima (TI) was very thin and composed of a continuous layer of thin, flattened endothelial cells with flattened nuclei. The luminal surface of the aorta was very regular and smooth. The tunica media (TM) was characterized by numerous diverse elastic laminae, which were wavy, parallel, and regularly arranged. Smooth muscle cells (SMCs) with oval to flattened nuclei were regularly arranged in the narrow spaces between the concentric lamellae. The tunica adventitia (TA) exhibited a normal morphology and did not show any fibrosis (Figure 3A).

In contrast, the aorta of aging rats exhibited noticeable degenerative morphology. The TI was thick to the extent that it could easily be distinguished from the TM. The endothelial lining was marked by endothelial cell proliferation, as their nuclei were close to each other with invasion of the sub-endothelial connective tissues, resulting in a relatively rough and irregular luminal surface. The TM was particularly thick with an increased number of elastic lamellae. The elastic fibers of the TM were thin and remarkably spacious, with some degree of fragmentation. SMCs were also proliferating between the elastic laminae and exhibited binucleate and multinucleate appearances. The TA was thickened and showed fibrosis (Figure 3B). Aortic wall thickness showed a significant increase in aging rats compared to young rats (Figure 3C).

### 3.4 Aging alters nuclear morphology

**Figure 4:**
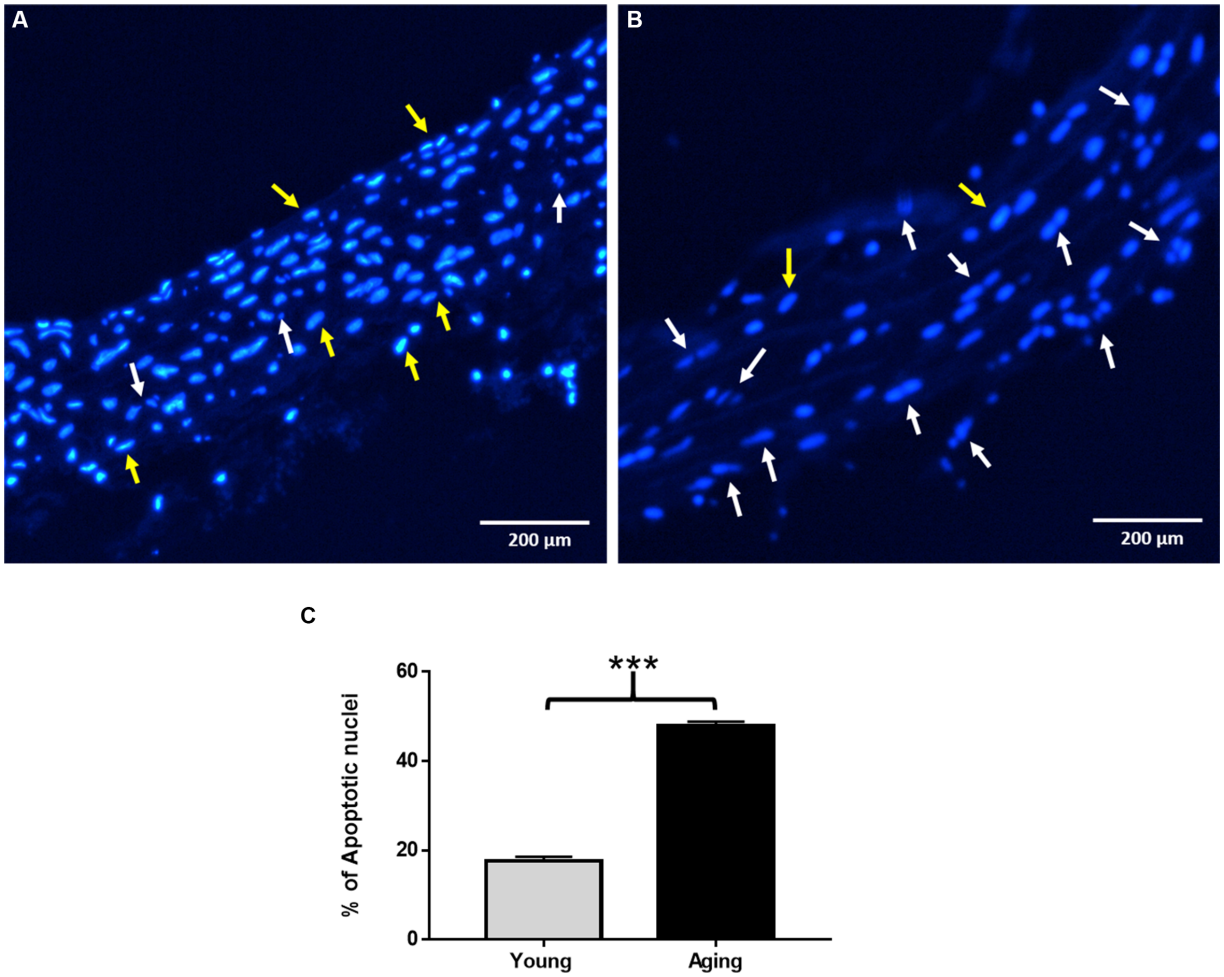
Assessment of nuclear morphological changes by Hoechst 33258 staining. **(A)** Representative image showing normal nuclear morphology in young rats. **(B)** Representative image showing typical apoptotic nuclear morphology such as chromatin condensation, nuclear fragmentation, and increased number of apoptotic nuclei in aging rats. Scale bar = 200 μm. **(C)** % of apoptotic nuclei significantly increased in aging rats ^***^p < 0.001 vs. young rats. The yellow arrow points to normal nuclei, White arrow points to apoptotic nuclei.

Nuclear morphological changes in the aortic tissues were analyzed using Hoechst 33258 staining. As shown in Figure 4A, the nucleus exhibited a normal and intact morphology with fewer apoptotic nuclei in young rats. In contrast, aging rats displayed typical apoptotic morphological changes, including chromatin condensation, nuclear fragmentation, and a higher number of apoptotic bodies, as observed in Figure 4B. The percentage of apoptotic nuclei in aging rats was significantly higher than that in young rats, as illustrated in Figure 4C.

### 3.5 Lipid accumulation in the aortic wall increases with age

**Figure 5:**
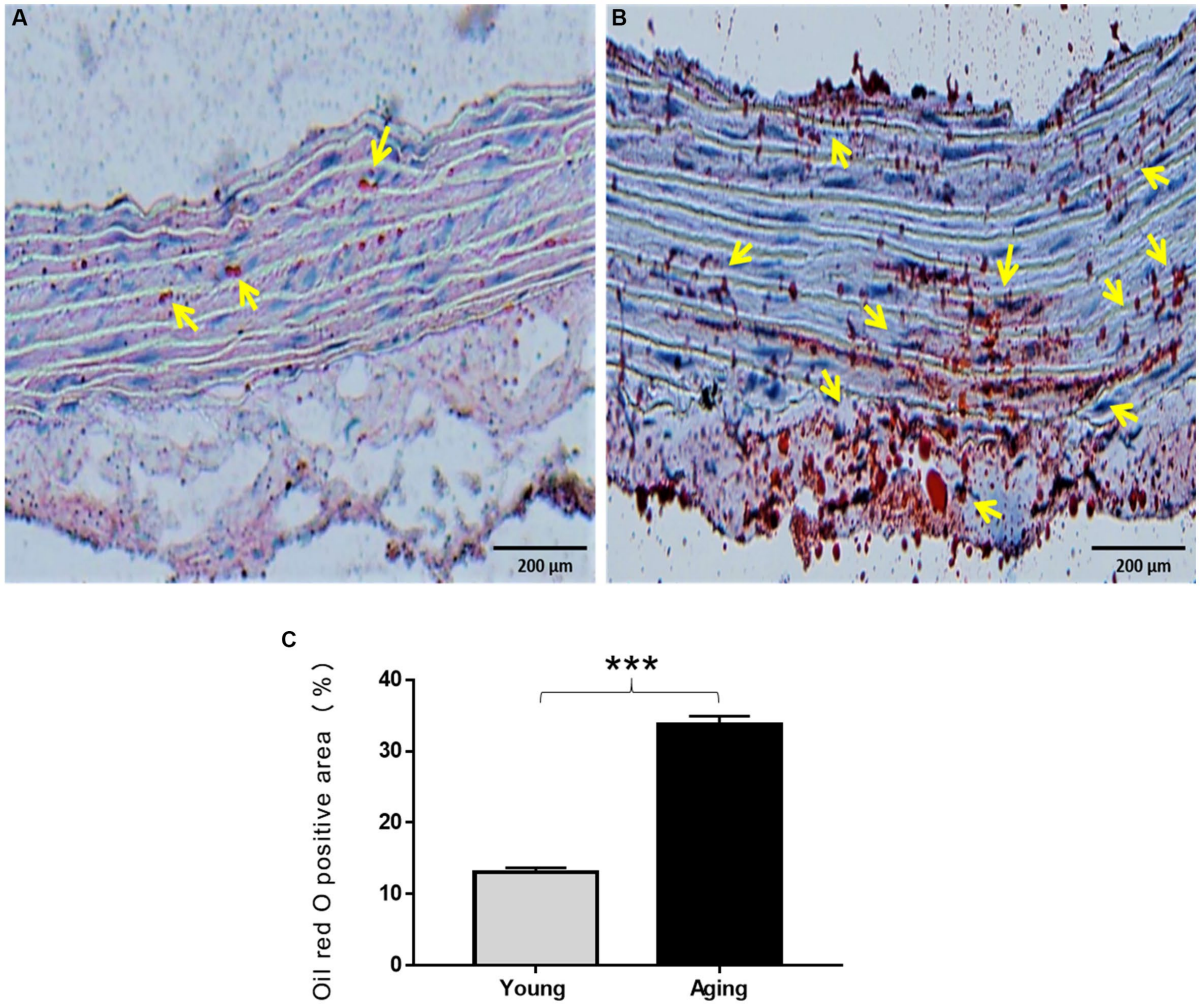
Oil Red O staining of the aorta of the young and aging rats. **(A)** Representative images display weak ORO staining in the aorta of young rats **(B)** and high ORO staining in the aorta of aging rats which represents the accumulation of lipids in the aortic wall. **(C)** % of ORO positive area significantly increased in aging rats ^***^p < 0.001 vs. young rats (*n* = 3). ORO, Oil Red O; yellow arrow, lipid droplets appeared as red granules in the aorta. Scale bar = 200 μm.

Oil Red O (ORO) staining was performed to visualize the quantity and intracellular localization of neutral triglycerides, lipids, and fat contents in the aorta. As illustrated in Figure 5A, young rats displayed minimal ORO lipid staining, with only a few lipid droplets observed in the aortic wall. In contrast, aging rats exhibited strong ORO staining in the aorta. Under microscopic examination, the lipid droplets appeared as streaks of small red granules or grape-like clusters in all three layers. Additionally, some enlarged lipid droplets were detected in certain regions within the tunica media and tunica adventitia, indicating a high amount of fat deposition in those specific areas (Figure 5B). The percentage of ORO-positive area was significantly higher in the aorta of aging rats (Figure 5C).

### 3.6 Altered expression of antioxidant proteins with aging in the aorta

**Figure 6:**
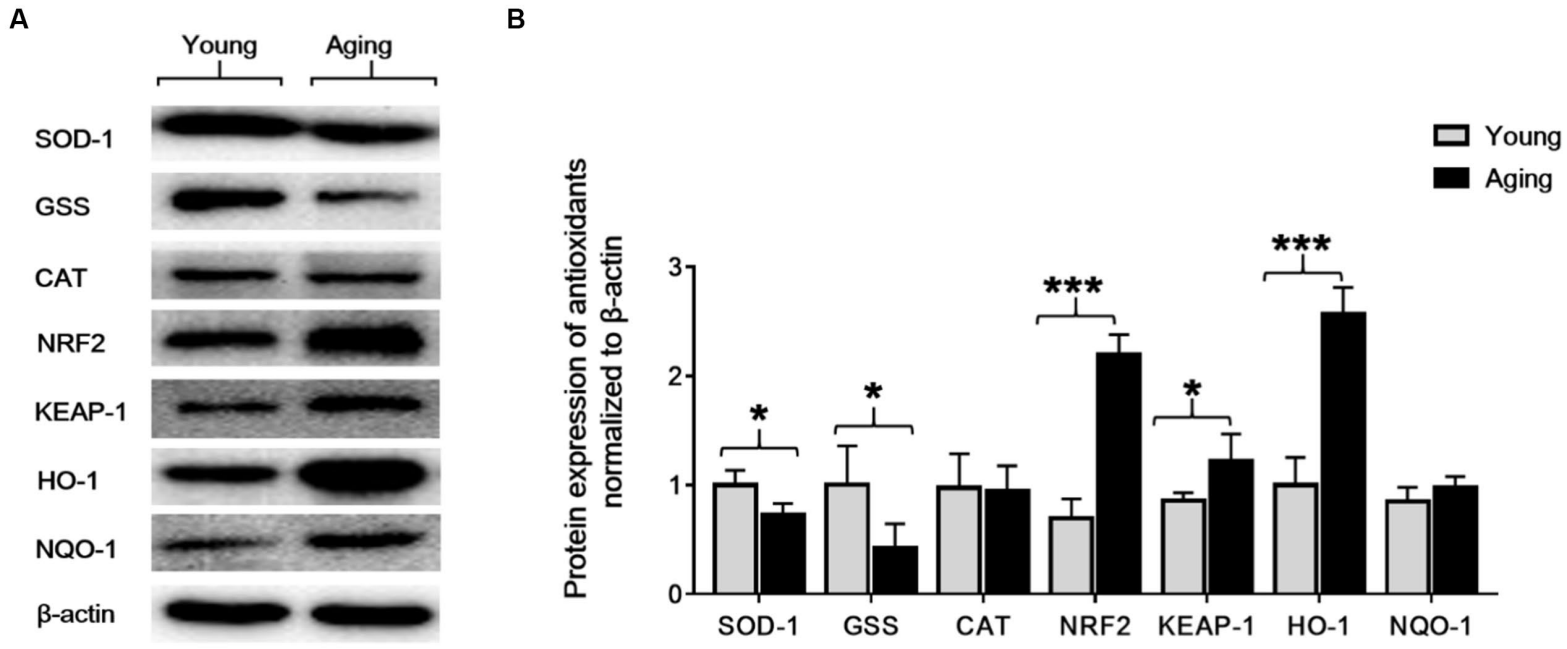
The protein expression levels of SOD-1, GSS, CAT, NRF2, KEAP-1, HO-1, and NQO-1 in the aorta of young and aging rats. **(A)** Representative immunoblots. **(B)** Densitometric analysis shows that the protein expression levels of SOD-1 and GSS significantly decreased ^*^p < 0.05, whereas NRF2, KEAP-1, and HO-1 significantly increased ^***^p < 0.001, ^*^p < 0.05, ^***^p < 0.001, respectively, in the aging rats compared to the young rats. β-actin was used as an internal control. SOD-1, superoxide dismutase-1; GSS, glutathione synthetase; CAT, catalase; NRF2, nuclear factor-erythroid 2-related factor-2; KEAP-1, Kelch-like ECH-associated protein; HO-1, heme oxygenase-1; NQO-1, NAD(P)H: quinone oxidoreductase.

Altered expression of antioxidant proteins with aging in the aorta. To investigate the effect of aging on the aorta’s antioxidant enzymes, we analyzed protein expression levels of SOD-1, CAT, GSS, NRF2, KEAP-1, HO-1, and NQO-1 by western blot. Western blot results revealed that, compared to young rats, the protein expression levels of SOD-1 and GSS decreased significantly, whereas the protein expression levels of NRF2, KEAP-1, and HO-1 proteins significantly increased in aging rats. However, the protein expression of CAT was slightly decreased, and the protein expression of NQO-1 was slightly increased in aging rats compared to young rats (Figures 6A,B).

**Figure 7:**
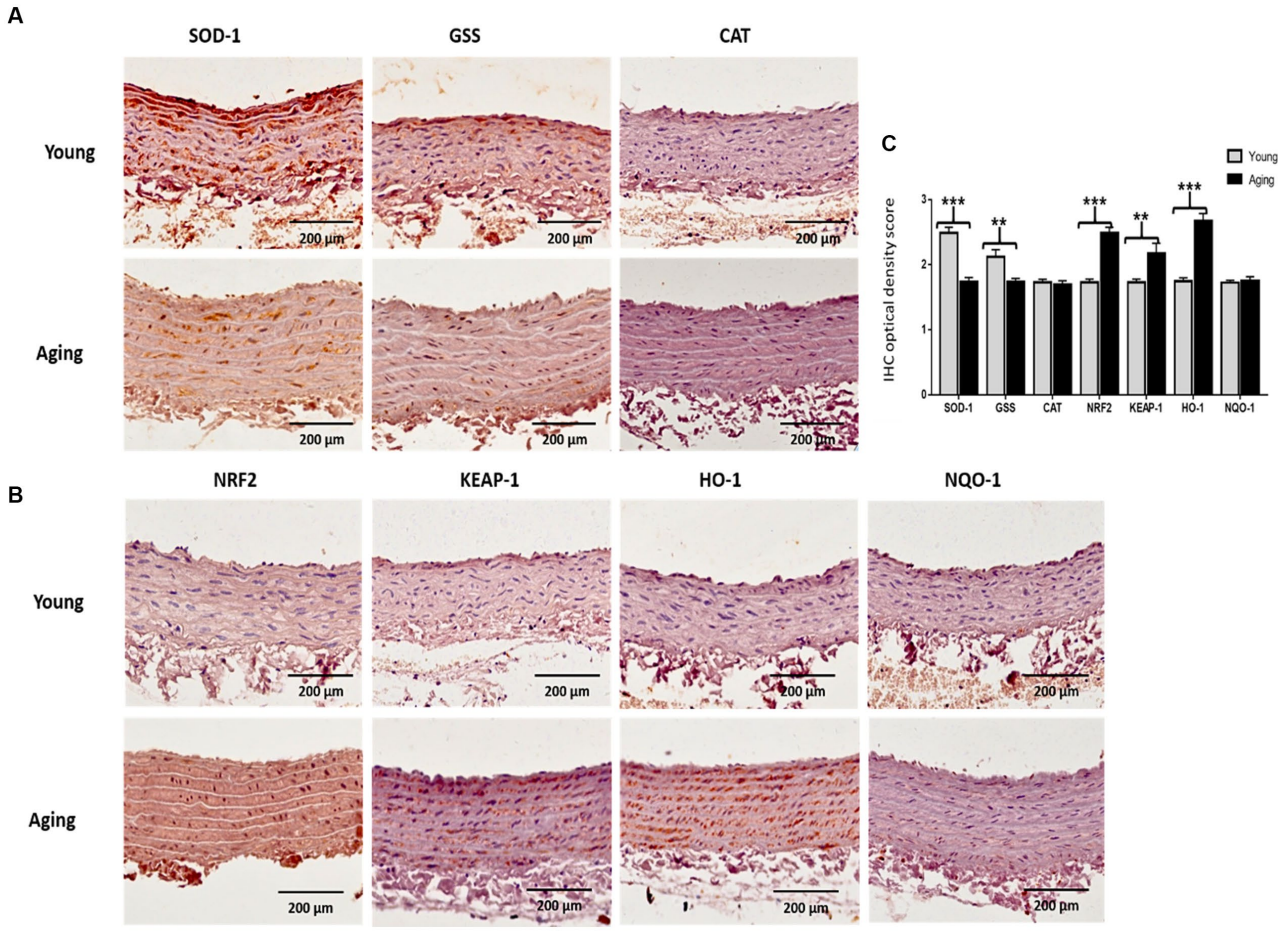
**(A)** Representative images of immunohistochemically stained slices for SOD-1, GSS, and CAT **(B)** as well as for NRF2, KEAP-1, HO-1, and NQO-1. The brownish color indicates positive immunostaining. Scale bar = 200 μm. **(C)** The mean optical intensity density (MOID) showed a significant decrease in SOD-1 and GSS (^***^p < 0.001, ^**^p < 0.01, respectively), whereas MOID of NRF2, KEAP-1, and HO-1 significantly increased (^***^p < 0.001, ^**^p < 0.01, ^***^p < 0.001, respectively) in aging rat, compared with young rats. However, CAT and NQO-1 remained unchanged in the two groups (no statistical significance). IHC, Immunohistochemistry; OD, Optical density.

Immunohistochemistry (IHC) was performed to visualize the expression of antioxidant proteins in the aorta of young and aging rats. IHC results confirmed the Western blot findings and revealed that the protein expression levels of SOD-1 and GSS decreased significantly in aging rats. However, in contrast, the immunostaining for NRF2, KEAP-1, and HO-1 increased significantly in the aorta of aging rats compared to young rats. Notably, immunostaining for CAT and NQO-1 was weak in both groups, and no noticeable difference was observed between the young and aging rats (Figures 7A,B). Quantitative assessments of the staining confirmed that the mean optical intensity density (MOID) score for SOD-1 and GSS showed a significant decrease, while the score for NRF2, KEAP-1, and HO-1 showed a significant increase in aging rats relative to the younger group. The MOID score for CAT and NQO-1 did not differ significantly in both groups (Figure 7C).

## 4 Discussion

The present study evaluated the association between vascular aging and the antioxidant defense system by investigating age-induced alterations in blood pressure, serum lipid levels, histological analysis, and antioxidant capacity in the aorta of young and aging rats. Various studies have reported the rise in blood pressure, which is considered an unavoidable part of healthy aging and is predominantly accompanied by structural alterations in the arteries (27). Our results, showing a significant elevation in SBP, DBP, and PP in our aging model, align with previous data. Additionally, previous data have reported that high blood pressure is associated with an imbalance between oxidants and antioxidants (28). Notably, the correlation between the age-associated decline in circulating sex hormones and elevated blood pressure has also been documented (29). These findings, along with our data, suggest that there is a significant correspondence between an age-related rise in blood pressure, which could result from aortic structural changes and variations in antioxidant status.

It is well-known that aging affects normal cholesterol metabolism, resulting in an alteration of the serum lipid profile (30). This study detected significantly higher serum LDL levels and significantly lower serum HDL levels in aging rats compared to young rats. However, no significant changes in serum TG and TC levels were observed with aging. Consistent with our findings, previous reports have also mentioned age-related higher LDL and lower HDL levels (31, 32). Likewise, no significant changes in TG and TC levels were observed between the 24-month-old and 10-month-old rats (33). It is well acknowledged that high LDL/low HDL levels in the blood contribute to the accumulation of fat in the aorta and increase the incidence of atherosclerosis (10). We further examined whether these age-related alterations in serum cholesterol levels are associated with the accumulation of fat/lipids in the aorta. As expected, we observed high lipid accumulation in the aged aorta. Notably, we did not observe any apparent atheromatous plaque in the aorta of aging rats. These findings suggest that the increased fat/lipid components in the aorta might be due to altered blood cholesterol levels and can be considered a normal part of aging, independent of atherosclerosis.

Previous studies have reported that the aging process results in notable structural and histological changes within the aortic wall (1, 3). In our examination of histological changes in the aortas of both young and aging rats using H&E staining, we found that the basic architecture of the aortic wall is altered in aging rats. In comparison, aortas obtained from young rats exhibited a normal aortic morphology. These observations in the aorta of aging rats are consistent with previous reports using different aging models (34, 35). Similar structural alterations in the aorta were also observed in aged male albino rats, supporting our findings (4). In humans, age-associated modifications in vascular structure have been reported as well (36, 37). Furthermore, we conducted an investigation into the effects of aging on nuclear morphology and apoptosis. Results from Hoechst staining revealed that the aortas of aging rats exhibited typical apoptotic nuclear features such as blebbing, nuclear fragmentation, chromatin condensation, and cell shrinkage. Notably, the percentage of apoptotic nuclei was significantly higher in the aged aorta, which aligns with previously reported findings (6). Importantly, the increased apoptosis observed during aging is considered, in one instance, as a protective mechanism of the body against the accumulation of injurious defective cells. In another context, it is related to an age-related decline in the structure and functional integrity of various tissues (6).

Data from various studies have described that the accumulation of oxidative damage, resulting from an imbalance between oxidants and antioxidants, might play a crucial role in the structural alterations and increased apoptosis observed in various tissues during the aging process (6, 8). These findings lead us to hypothesize that the physiological aging process, which is accompanied by degenerative structural changes and increased apoptosis in the aorta, could involve oxidative mechanisms. However, the underlying mechanism in the aged aorta remains poorly understood.

Antioxidant enzymes protect the body against the deleterious effect of oxidative stress, and a decline in the antioxidant capacity results in the production of oxidative stress-related cellular damage in the tissues (11). Previously, there were very limited studies that investigated the link between age-related alterations in the expression of antioxidant enzymes, particularly NRF2 activation and its downstream regulated proteins, in the aorta of aging rats. Most of these studies primarily focused on male rats (38) To assess the impact of aging on the aorta’s antioxidant capacity in female rats, we conducted an analysis and compared the expression levels of several antioxidative and cytoprotective proteins, including SOD-1, GSS, CAT, NRF2, KEAP-1, HO-1, and NQO-1, in both young and aging female rats. Western blot results and immunohistochemical analyses revealed that in the aorta of aging rats, SOD-1 and GSS significantly decreased, while NRF2, KEAP-1, and HO-1 significantly increased in comparison with young rats. Additionally, the protein expression levels of CAT and NQO-1 remained unaltered. These results suggest that the age-related decline in the expression of SOD-1 and GSS could account for oxidative stress-induced cellular damage to the aortic cells, leading to morphological and physiological alterations in the aorta with senescence. Similar to our findings, a study was performed in male Wistar rats with ages ranging from 1 month to 24 months. The findings demonstrated an age-related decline in SOD-1, while CAT expression remained unchanged in the aorta (39). We could not find any research related to GSS alteration with aging in the aorta. However, a few studies have reported changes in GSS protein expression in different organs with aging (40). Surprisingly, the noticeable increase in the basal expressions of NRF2, KEAP-1, and HO-1 proteins in the aorta of aging rats is supported by the findings of others in various organs other than the aorta (41). Zhou et al. found that the basal expression of NRF2-regulated genes increased with aging in human bronchial epithelial cells, while inducible expression declined in these cells (42). Contrary to our findings, several studies have reported impaired KEAP-1/NRF2 signaling and down-regulation of both NQO-1 and HO-1 with aging (43). For instance, Ungvari et al. reported that vascular oxidative stress is accompanied by decreased expression of NRF2, NQO-1, and HO-1 in aged Fischer 344—Brown Norway rats (44). This inconsistency in results may be attributed to variations in the types of cells, tissues, and animal models used. Further investigations are required to clarify these contradictory findings. Recent studies have highlighted that when eukaryotic cells are exposed to oxidative stress, they activate a complex redox-sensitive NRF2 antioxidant signaling pathway. This pathway serves to protect cells against oxidative injury and maintain cellular homeostasis (13). The NRF2 signaling pathway up-regulates various antioxidants and phase II detoxifying enzymes, including HO-1 and NQO-1, which are essential components of the cellular stress response and play a critical role in protecting cells from oxidative insults (43). Importantly, NRF2 is primarily located in the nucleus of aortic cells, suggesting the potential basal induction of the NRF2 signaling pathway in the aorta in response to oxidative stress that occurs during aging. These findings indicate that the aorta in young rats does not experience cellular injury or oxidative damage, as evidenced by the basal levels of the NRF2 signaling pathway and its subsequent downstream regulation of NQO-1 and HO-1 proteins. However, in aging rats, increased levels of NRF2 and KEAP-1 proteins in aortic tissues explain the age-associated overactivation of the NRF2 signaling pathway. Furthermore, the increased expression of HO-1 in the aorta of aging rats confirms that the induction of this enzyme is governed by the activation of the NRF2 pathway in response to oxidative stress in aortic tissues. Interestingly, we found that the expression of NQO-1 protein was not significantly different in young and aging rats. This suggests that, in the aging aorta of female SD rats, the transcriptional antioxidant response of the NRF2 pathway primarily contributes to the up-regulation of HO-1, rather than NQO-1. Recent academic studies have focused on the activation of HO-1 through the NRF2 pathway in the context of various diseases. These studies have revealed that this pathway acts as a protective response to cellular injury caused by oxidative stress. Furthermore, empirical evidence has supported the pathway’s effectiveness in reducing oxidative stress-induced vascular injuries and atherosclerosis (45–47). Building upon these findings and our own research, it is expected that in the process of healthy aging, the natural activation of the NRF2 pathway will increase the expression of HO-1 in the aorta when confronted with oxidative stress. This response is anticipated to function as a compensatory mechanism in the preservation of healthy aging. Further investigations, however, are required to fully elucidate the fundamental mechanisms involved in the activation of HO-1 by the NRF2 pathway and its regulation in the aged aorta, which can be achieved through knockdown or overexpression studies.

In summary, there is a substantial correlation between degenerative histological changes and decreased antioxidant capacity in the aorta during the process of physiological aging. The degenerative structural changes associated with aging may result from oxidative stress caused by reduced tissue production of key antioxidants, such as SOD-1 and GSS. In response, eukaryotic cells may activate the NRF2 signaling pathway and increase HO-1 enzyme production as a compensatory strategy to preserve normal aortic function in the aging aorta. This promotes healthy aging in the absence of antecedent pathological illness and can be investigated as a novel therapeutic target for various age-related cardiovascular disorders.

## 5 Limitations

This study has limitations. Firstly, it concentrated on the aorta and its antioxidant defense mechanisms, which may limit the generalizability of the findings to other bodily tissues and organs. Secondly, the analysis was restricted to aortic aging at a fixed time point (24 months in rats), and a more comprehensive understanding of the aging process would require examining aortic changes across various aging stages. Finally, our study hinted at the activation of the NRF2/HO-1 pathway but did not explore the detailed mechanisms governing this pathway. Future research should aim to provide a more comprehensive understanding of its role in the aging process.

## 6 Conclusion

In this study, we investigated age-induced aortic modifications in female rats and their association with alterations in the antioxidant defense system. Specifically, we explored the potential activation of HO-1 through the NRF2 signaling pathway, which is expected to function as an adaptive response, protecting against oxidative damage and promoting healthy aging. These findings provide insight into the role of the NRF2/HO-1 pathway in aortic aging and contribute to our understanding of the aging process. However, further research is needed to fully comprehend the complexities of this signaling pathway within the aging aorta and its potential as a novel therapeutic target for age-related disorders.

## Data availability statement

The raw data supporting the conclusions of this article will be made available by the authors, without undue reservation.

## Ethics statement

The current research was approved by the Harbin Medical University, Harbin, China. Notably, all animal experimentation was conducted in accordance with applicable laws, regulations, and guidelines, prioritizing animal welfare and minimizing any potential harm. The study was conducted in accordance with the local legislation and institutional requirements.

## Author contributions

SBA: Conceptualization, Data curation, Formal analysis, Investigation, Methodology, Software, Validation, Visualization, Writing – original draft, Writing – review & editing. XQ: Conceptualization, Investigation, Methodology, Visualization, Writing – original draft. JW: Investigation, Methodology, Visualization, Writing – original draft. WA: Validation, Writing – review & editing. QG: Data curation, Visualization, Writing – original draft. YC: Methodology, Visualization, Writing – original draft. YH: Methodology, Visualization, Writing – original draft. YB: Funding acquisition, Writing – review & editing. TA: Writing – review & editing. GW: Writing – review & editing. MB: Writing – review & editing. CL: Conceptualization, Project administration, Resources, Supervision, Writing – original draft. HZ: Conceptualization, Formal analysis, Project administration, Funding acquisition, Resources, Software, Supervision, Validation, Writing – review & editing.
